# Loss of brain inter-frequency hubs in Alzheimer's disease

**DOI:** 10.1038/s41598-017-07846-w

**Published:** 2017-09-07

**Authors:** J. Guillon, Y. Attal, O. Colliot, V. La Corte, B. Dubois, D. Schwartz, M. Chavez, F. De Vico Fallani

**Affiliations:** 1 Inria Paris, Aramis project-team, 75013 Paris, France; 2 CNRS UMR-7225, Sorbonne Universites, UPMC Univ Paris 06, Inserm U-1127, Institut du cerveau et la moelle (ICM), Hopital Pitie-Salpetriere, 75013 Paris, France; 3 MyBrain Technologies, Paris, France; 4 Department of Neurology, Institut de la Memoire et de la Maladie dAlzheimer - IM2A, Paris, France; 5 Institute of Psychology, University Paris Descartes, Sorbonne Paris Cite, France; 6 INSERM UMR 894, Center of Psychiatry and Neurosciences, Memory and Cognition Laboratory, Paris, France

## Abstract

Alzheimer’s disease (AD) causes alterations of brain network structure and function. The latter consists of connectivity changes between oscillatory processes at different frequency channels. We proposed a multi-layer network approach to analyze multiple-frequency brain networks inferred from magnetoencephalographic recordings during resting-states in AD subjects and age-matched controls. Main results showed that brain networks tend to facilitate information propagation across different frequencies, as measured by the multi-participation coefficient (*MPC*). However, regional connectivity in AD subjects was abnormally distributed across frequency bands as compared to controls, causing significant decreases of *MPC*. This effect was mainly localized in association areas and in the cingulate cortex, which acted, in the healthy group, as a true inter-frequency hub. *MPC* values significantly correlated with memory impairment of AD subjects, as measured by the total recall score. Most predictive regions belonged to components of the default-mode network that are typically affected by atrophy, metabolism disruption and amyloid-*β* deposition. We evaluated the diagnostic power of the *MPC* and we showed that it led to increased classification accuracy (78.39%) and sensitivity (91.11%). These findings shed new light on the brain functional alterations underlying AD and provide analytical tools for identifying multi-frequency neural mechanisms of brain diseases.

## Introduction

Recent advances in network science has allowed new insights in the brain organization from a system perspective. Characterizing brain networks, or connectomes, estimated from neuroimaging data as graphs of connected nodes has not only pointed out important network features of brain functioning - such as small-worldness, modularity, and regional centrality - but it has also led to the development of biomarkers quantifying reorganizational mechanisms of disease^1^. Among others, Alzheimer’s disease (AD), which causes progressive cognitive and functional impairment, has received great attention by the network neuroscience community^1–3^. AD is histopathologically defined by the presence of amyloid-*β* plaques and tau-related neurofibrillary tangles, which cause loss of neurons and synapses in the cerebral cortex and in certain subcortical regions^2^. This loss results in gross atrophy of the affected regions, including degeneration in the temporal and parietal lobe, and parts of the frontal cortex and cingulate gyrus^4^.

Structural brain networks, whose connections correspond to inter-regional axonal pathways are therefore directly affected by AD because of connectivity disruption in several areas including cingulate cortices and hippocampus^5, 6^. A decreased number of fiber connections eventually lead to a number of network changes on multiple topological scales. At larger scales, AD brain networks estimated from diffusion tensor imaging (DTI) showed increased characteristic path length as compared to healthy subjects leading to a global loss of network small-worldness^2, 7^. Similar topological alterations have been also documented in resting-state brain networks estimated from functional magnetic resonance imaging (fMRI)^8^, as well as from magneto/electroencephalographic (M/EEG) signals, the latter ones often reported within the *alpha* frequency range (8–13 Hz) which is typically affected in AD^9–11^. On smaller topological scales, structural brain network studies have demonstrated a loss of connector hubs in temporal and parietal areas that correlates with cognitive decline^2, 12, 13^. In addition, higher-order association regions appear to be affected in functional brain networks inferred from fMRI^2, 14^ and MEG signals, the latter showing a characteristic loss of parietal hubs in higher (>14 Hz) frequency ranges^15, 16^.

Graph analysis of brain networks has advanced our understanding of the organizational mechanisms underlying human cognition and disease, but a certain number of issues still remain to be addressed^17, 18^. For example, conventional approaches analyze separately brain networks obtained at different frequency bands, or in some cases, they simply focus on specific frequencies, thus neglecting possible insights of other spectral contents on brain functioning^17^. However, several studies have hypothesized and reported signal interaction or modulations between different frequency bands that are supportive of cognitive functions such as memory formation^19–21^. Moreover, recent evidence shows that neurodegenerative processes in AD do alter functional connectivity in different frequency bands^16, 22, 23^. How to characterize this multiple information from a network perspective still remains poorly explored. Here, we proposed a multi-layer network approach to study multi-frequency connectomes, where each layer contains the brain network extracted at different bands. Multi-layer network theory has been previously used to synthesize MEG connectomes from a whole population^24^, characterize temporal changes in dynamic fMRI brain networks^12^, and integrating structural information from multimodal imaging (fMRI, DTI)^25, 26^. Its applicability to multi-frequency brain networks has been recently illustrated in fMRI connectomes for which, however, the frequency ranges of interest remains quite limited^27^.

We focused on source-reconstructed MEG connectomes, characterized by rich frequency dynamics, that were obtained from a group of AD and control subjects in eyes-closed resting-state condition. We hypothesized that the atrophy process in AD would lead to an altered distribution of regional connectivity across different frequency bands and we used the multi-participation coefficient to quantify this effect both at global and local scale^28^. We evaluated the obtained results, which provide a novel view of the brain reorganization in AD, with respect to standard approaches based on single-layer network analysis and flattening schemes^29^. Finally, we tested the diagnostic power of the measured brain network features to discriminate AD patients and healthy subjects.

## Methods

### Experimental design and data pre-processing

The study involved 25 Alzheimer’s diseased (AD) patients (13 women) and 25 healthy age-matched control (HC) subjects (18 women). All participants underwent the Mini-Mental State Examination (MMSE) for global cognition^30^ and the Free and Cued Selective Reminding Test (FCSRT) for verbal episodic memory^31–33^. Specifically, we considered the Total Recall (TR) score - given by the sum of the free and cued recall scores - which has been demonstrated to be highly predictive of AD^34^ (Table 1).

**Table 1:** Characteristics, cognitive and memory scores of experimental subjects.

	Control (HC)	Alzheimer (AD)	*p*-value
Age	70.8 (9.1)	73.5 (9.4)	0.3142
MMSE	28.2 (1.4)	23.2 (3.6)	<10^−5^
FR	31.5 (6.6)	14.9 (6.5)	<10^−5^
TR	46.3 (1.5)	33.9 (10.0)	<10^−5^

Mean values and standard deviations (between parentheses) are reported. The last column shows the *p*-values returned by a non-parametric permutation t-tests with 10000 realizations. MMSE = mini-mental state examination score; TR = total recall memory test score (/48); FR = free recall memory test (/48).

Inclusion criteria for all participants were: *i)* age between 50 and 90; *ii)* absence of general evolutive pathology; *iii)* no previous history of psychiatric diseases; *iv)* no contraindication to MRI examination; *v)* French as a mother tongue. Specific criteria for AD patients were: *i)* clinical diagnosis of Alzheimer’s disease; *ii)* Mini-Mental State Examination (MMSE) score greater or equal to 18. Magnetic resonance imaging (MRI) acquisitions were obtained using a 3T system (Siemens Trio, 32-channel system, with a 12-channel head coil). The MRI examination included a 3D T1-weighted volumetric magnetization-prepared rapid-gradient echo (MPRAGE) sequence with 1 mm isotropic resolution and the following parameters: repetition time (TR) = 2300 ms, echo time (TE) = 4.18 ms, inversion time (TI) = 900 ms, matrix = 256 × 256. This sequence provided a high contrast-to-noise ratio and enabled excellent segmentation of high grey/white matter.

The magnetoencephalography (MEG) experimental protocol consisted in a resting-state with eyes-closed (EC). Subjects seated comfortably in a dimly lit electromagnetically and acoustically shielded room and were asked to relax. MEG signals were collected using a whole-head MEG system with 102 magnetometers and 204 planar gradiometers (Elekta Neuromag TRIUX MEG system) at a sampling rate of 1000 Hz and on-line low-pass filtered at 330 Hz. The ground electrode was located on the right shoulder blade. An electrocardiogram (EKG) Ag/AgCl electrodes was placed on the left abdomen for artifacts correction and a vertical electrooculogram (EOG) was simultaneously recorded. Four small coils were attached to the participant in order to monitor head position and to provide co-registration with the anatomical MRI. The physical landmarks (the nasion, the left and right pre-auricular points) were digitized using a Polhemus Fastrak digitizer (Polhemus, Colchester, VT).

We recorded three consecutive epochs of approximately 2 minutes each. All subjects gave written informed consent for participation in the study, which was approved by the local ethics committee of the Pitie-Salpetriere Hospital. All experiments were performed in accordance with relevant guidelines and regulation. Signal space separation was performed using MaxFilter^35^ to remove external noise. We used in-house software to remove cardiac and ocular blink artifacts from MEG signals by means of principal component analysis. We visually inspected the preprocessed MEG signals in order to remove epochs that still presented spurious contamination. At the end of the process, we obtained a coherent dataset consisting of three clean preprocessed epochs for each subject.

### Source reconstruction, power spectra and brain connectivity

We reconstructed the MEG activity on the cortical surface by using a source imaging technique^36, 37^. We used the FreeSurfer 5.3 software (surfer.nmr.mgh.harvard.edu) to perform skull stripping and segment grey/white matter from the 3D T1-weighted images of each single subject^38, 39^. Cortical surfaces were then modeled with approximately 20000 equivalent current dipoles (i.e., the vertices of the cortical meshes). We used the Brainstorm software^40^ to solve the linear inverse problem through the wMNE (weighted Minimum Norm Estimate) algorithm with overlapping spheres^41^. Both magnetometer and gradiometer, whose position has been registered on the T1 image using the digitized head points, were used to localize the activity over the cortical surface. The reconstructed time series were then averaged within 148 regions of interest (ROIs) defined by the Destrieux atlas^42^.

We computed the power spectral density (PSD) of the ROI signals by means of the Welch’s method; we chose a 2 seconds sliding Hanning window, with a 25% overlap. The number of FFT points was set to 2000 for a frequency resolution of 0.5 Hz. We estimated functional connectivity by calculating the spectral coherence (Supplementary Text) between each pair of ROI signals^43^. As a result, we obtained for each subject and epoch, a set of connectivity matrices of size 148 × 148 where the (*i*, *j*) entry contains the value of the spectral coherence between the signals of the ROI *i* and *j* at a frequency *f* = 0,0.5, …, 499.

We then averaged the connectivity matrices within the following characteristic frequency bands^44, 45^: *delta* (2–4 Hz), *theta* (4.5–7.5 Hz), *alpha1* (8–10.5 Hz), *alpha2* (11–13 Hz), *beta1* (13.5–20 Hz), *beta2* (20.5–29.5 Hz) and *gamma* (30–45 Hz). We finally averaged the connectivity matrices across the three available epochs to obtain a robust estimate of the individual brain networks whose nodes were the ROIs (*n* = 148) and links, or edges, were the spectral coherence values.

### Single-layer network analysis

In order to cancel the weakest noisy connections, we thresholded and binarized the values in the connectivity matrices. Specifically, we retained the same number of links for each brain network. We considered six representative connection density thresholds corresponding to an average node degree *k* = {1, 3, 6, 12, 24, 48}. These values cover the density range [0.007, 0.327] which contains the typical density values used in complex brain network analysis^17, 18, 46^. The resulting sparse brain networks, or graphs, were represented by adjacency matrices *A*, where the *a*_*ij*_ entry indicates the presence or absence of a link between nodes *i* and *j*.

#### Participation coefficient

Given a network partition, the local participation coefficient (*PC*_*i*_) of a node *i* measures how evenly it is connected to the different clusters, or modules of the network^47^. Nodes with high participation coefficients are considered as central hubs as they allow for information exchange among different modules. The global participation coefficient *PC* of a network at layer *λ* is then given by the average of the *PC*_*i*_ values:

1 $$P{C}^{[\lambda ]}=\frac{1}{n}\sum _{i=1}^{N}P{C}_{i}^{[\lambda ]}=\frac{1}{n}\sum _{i=1}^{N}\,[1-\sum _{m=1}^{{M}^{[\lambda ]}}{(\frac{{k}_{i,m}^{[\lambda ]}}{{k}_{i}^{[\lambda ]}})}^{2}],$$

where ${k}_{i,m}^{[\lambda ]}$ is the number of links from the node *i* to the nodes of the module *m* in layer *λ* and ${k}_{i}^{[\lambda ]}$ is the degree of node *i* in layer *λ*. By construction, *PC* ranges from 0 to 1. Here, the partition of the networks into modules was obtained by maximizing the modularity function^48^.

#### Flattened networks

We also computed the participation coefficients for brain networks obtained by flattening the frequency layers into a single *overlapping* or *aggregated* network^28^. In an overlapping network, the weight of an edge *o*_*ij*_ corresponds to the number of times that the nodes *i* and *j* are connected across layers:

2 $${o}_{ij}=\sum _{\lambda }{a}_{ij}^{[\lambda ]},$$

In an aggregated network, the existence of an edge indicates that nodes *i* and *j* are connected in at least one layer:

3 $${a}_{ij}=\{\begin{array}{ll}1 & {\rm{if}}\,\exists \lambda :{a}_{ij}^{[\lambda ]}\ne 0\\ 0 & {\rm{otherwise}}\end{array},$$

Notice that, by construction, flattened networks do not preserve the original connection density of the single layer networks.

### Multi-layer network analysis

**Figure 1:**
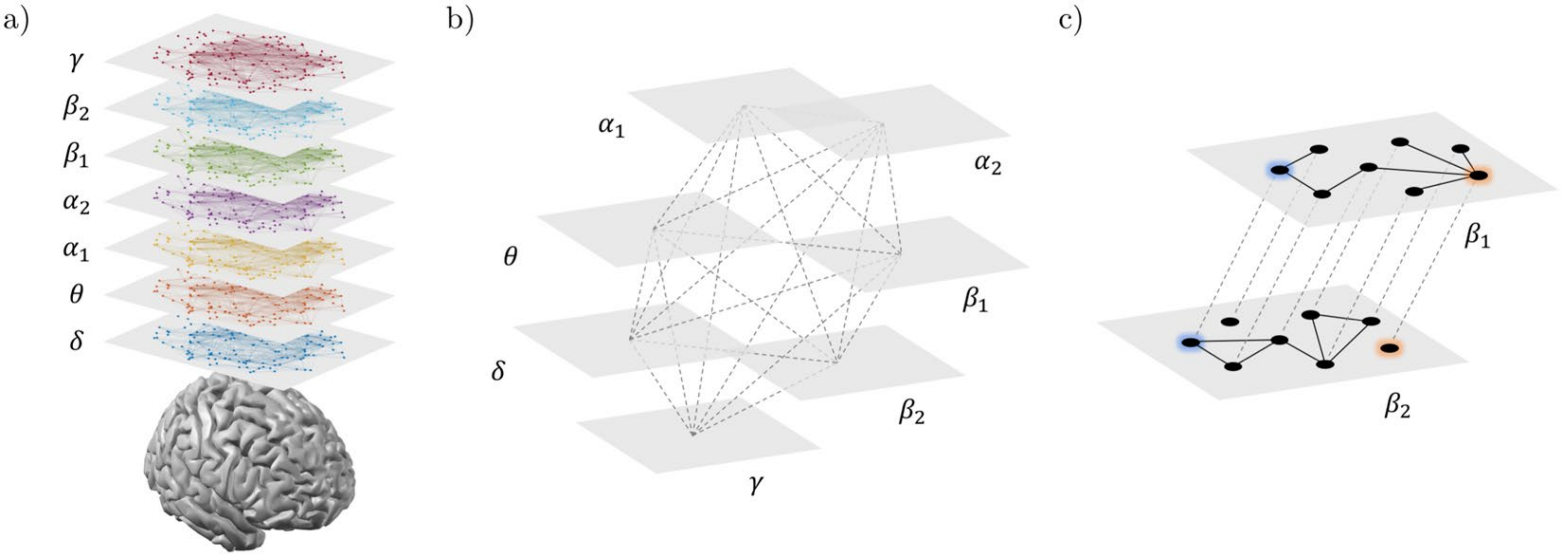
Multi-frequency brain networks. Panel (a) shows brain networks of a representative subject extracted from seven frequency bands. Links are inferred by means of spectral coherence and thresholded to have in each layer an average node degree *k* = 12. (**b**) Procedure to construct a multi-frequency network. Each layer corresponds to a different frequency band. Only nodes representing the same brain region in each layer are virtually connected. Hence, inter-layer links code for identity relationships. (**c**) Inter-frequency node centrality. A two-layer multiplex is considered for the sake of simplicity. The blue node acts as an inter-frequency hub (i.e., multi-participation coefficient *MPC* = 1) as it allows for a balanced information transfer between layer *β*_*1*_ and *β*_*2*_; the red node, who is disconnected in layer *β*_*2*_, blocks the information flow and has *MPC* = 0.

We adopted a multi-layer network approach to integrate the information from brain networks at different frequency bands, while preserving their original structure. Specifically, we built for each subject a multiplex network (Fig. 1a,b) where the different layers correspond to different frequency bands and each node in one layer is virtually connected to all its counterparts in the other layers^28, 29^.

Without loss of generality, the resulting supra-adjacency matrix *A* is given by the intra-layer adjacency matrices on the main diagonal:

4 $${\mathscr{A}}=\{{A}^{[\delta ]},{A}^{[\theta ]},{A}^{[{\alpha }_{1}]},{A}^{[{\alpha }_{2}]},{A}^{[{\beta }_{1}]},{A}^{[{\beta }_{2}]},{A}^{[\gamma ]}\},$$

where *A* ^[*λ*]^ corresponds to the brain network at the frequency *λ*. Notice that inter-layer adjacency matrices of multiplexes are intrinsically defined as identity matrices^49, 50^.

#### Multi-participation coefficient

We considered the local multi-participation coefficient *MPC*_*i*_, as an akin version of the local participation coefficient *PC*_*i*_, to measure how evenly a node *i* is connected to the different layers of the multiplex^28^. This way, nodes with high *MPC*_*i*_ are considered central hubs as they would allow for a better information exchange among different layers. The global multi-participation coefficient is then given by the average of the *MPC*_*i*_ values:

5 $$MPC=\frac{1}{n}\sum _{i=1}^{N}MP{C}_{i}=\frac{1}{n}\sum _{i=1}^{N}\frac{M}{M-1}[1-\sum _{\lambda }{(NL{P}_{i}^{[\lambda ]})}^{2}],$$

where $NL{P}_{i}^{[\lambda ]}={k}_{i}^{[\lambda ]}/{o}_{i}$ stands for *node-degree layer proportion*, which measures the tendency of the connectivity of a node *i* to concentrate in layer *λ*. By construction, if nodes tend to concentrate their connectivity in one layer, the global multi-participation coefficient tends to 0; on the contrary, if nodes tend to have the same number of connections in every layer, the *MPC* value tends to 1 (Fig. 1c). In the singular case where a node is disconnected in every layer, we assigned *MPC*_*i*_ = 0 to avoid indeterminate results.

From a statistical perspective, a random walker reaching a node with low *MPC*_*i*_ will jump with higher probability to layers where the node degree is higher, while it will tend to avoid layers with lower node degrees. On the contrary, if *MPC*_*i*_ is high, the random walker can jump with similar probability to any other layer, and this would facilitate the information passing (or communication) across all the layers.

We further used the standard coefficient of variation *CV*_*i*_ to measure the dispersion of the degree of a node *i* across layers. A global coefficient of variation *CV* is then obtained by averaging the *CV*_*i*_ values across all the nodes (Supplementary Text).

### Statistical analysis

We first analyzed network features on global topological scales in order to detect statistical differences between AD and HC subjects at the whole system level. Only for the network features that resulted significantly different at the global scale, we also assessed possible group-differences at the local scale of single nodes. This hierarchical approach allowed us to associate brain network differences at multiple topological scales^51^. We used a non-parametric permutation t-test, to assess statistical differences between groups, with a significance level of 0.05^52, 53^. The permutation test generated a set of 10000 surrogate data by randomly exchanging the group labels (i.e., AD or HC) of the brain network features. The *t*-statistic and *p*-value were then extracted from the simulated distributions. At the local scale, we performed a permutation test for each node separately. Due to the large number of tests (i.e., 148), we applied a correction for multiple comparisons by computing an adjusted version of the false discovery rate (FDR)^54^.

To test the ability of the significant brain network features to predict the cognitive/memory impairment of AD patients, we used the non-parametric Spearman’s correlation coefficient *R*. We set a significance level of 0.05 for the correlation of global network features, with a FDR correction in the case of multiple comparisons (local features).

### Classification

We used a classification approach to evaluate the discriminating power of the local brain network features which resulted significantly different in the AD and HC group. Because we did not know in advance which were the most discriminating features, we tested different combinations. In particular, for each local network feature, we first ranked the respective ROIs according to the *p*-values returned by the between-group statistical analysis (see previous section). For each subject *s*, we then tested different feature vectors obtained by concatenating, one-by-one, the values of the network features extracted from the ranked ROIs. The generic feature vector *c*_*s*_ reads:

6 $${c}_{s}=[{g}_{1},\ldots ,{g}_{k}]$$

where *g*_*k*_ is a generic local network feature and *k* is a rank that ranges from 1 (the most significant ROI) to the total number of significant ROIs. When different network features were considered (e.g., *PC* and *MPC*), we concatenated the respective *c*_*s*_ feature vectors allowing for all the possible combinations.

To quantify the separation between the feature vectors of AD and HC subjects, we used a Mahalanobis distance classifier. We applied a repeated 5-folds cross-validation procedure where we randomly split the entire dataset into a training set (80%) and a testing test (20%). This procedure was eventually iterated 10000 times in order to obtain more accurate classification rates. To assess the classification performance we computed the sensitivity (*Sens*), specificity (*Spec*) and accuracy (*Acc*), defined respectively as the percentage of AD subjects correctly classified as AD, the percentage of HC subjects classified as HC and the total percentage of subjects (AD and HC) properly classified. We also computed the receiver operating characteristic (ROC) curve and its area under the curve (AUC)^55^.

### Data availability

The Matlab code for the manipulation of multi-layer networks and the computation of the *MPC*, together with the connectivity matrices generated and analyzed in this study, are available at the Brain Network Toolbox repository (https://github.com/brain-network/bnt).

## Results

**Figure 2:**
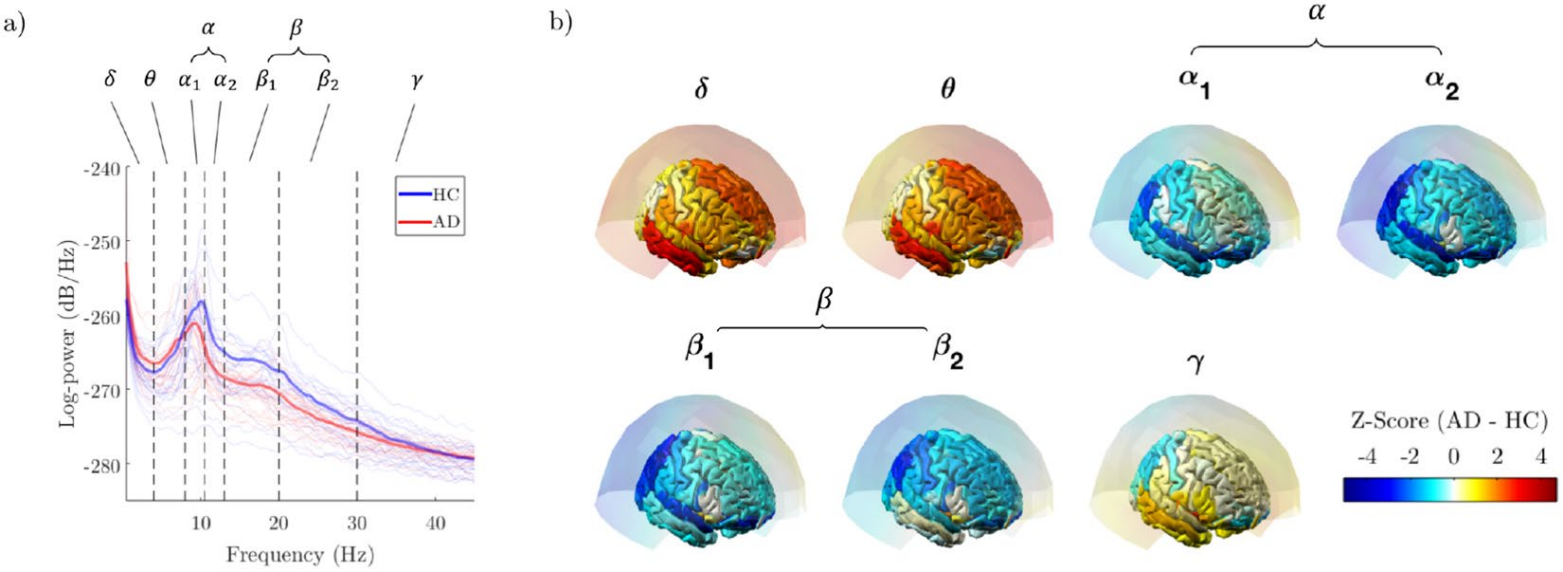
Spectral analysis of MEG signals. (**a**) Power spectrum density (PSD) for a representative occipital sensor before source reconstruction. Each line corresponds to a subject. Bold lines show the group-averaged values in the Alzheimer’s disease group (AD) and in the healthy control group (HC). (**b**) Statistical PSD group differences. Z-scores are obtained using a non-parametric permutation t-test. Results are represented both as sensor and source space.

Power analysis of source-reconstructed MEG signals confirmed the characteristic changes in the oscillatory activity of AD subjects compared to HC subjects (Fig. 2a)^56–59^. Significant *alpha* power decreases were more evident in the parietal and occipital regions (*Z* < −2.58), while significant *delta* power increases (*Z* > 2.58) were more localized in the frontal regions of the cortex (Fig. 2b).

### Reduced gamma inter-modular connectivity

As expected, the value of the connection density threshold had an impact on the network differences between groups. We selected the first threshold for which we could observe a significant group difference for both single- and multi-layer analysis. The obtained results determined the choice of a representative threshold, common to all the brain networks, corresponding to an average node degree *k* = 12 (Fig. S1).

**Figure 3:**
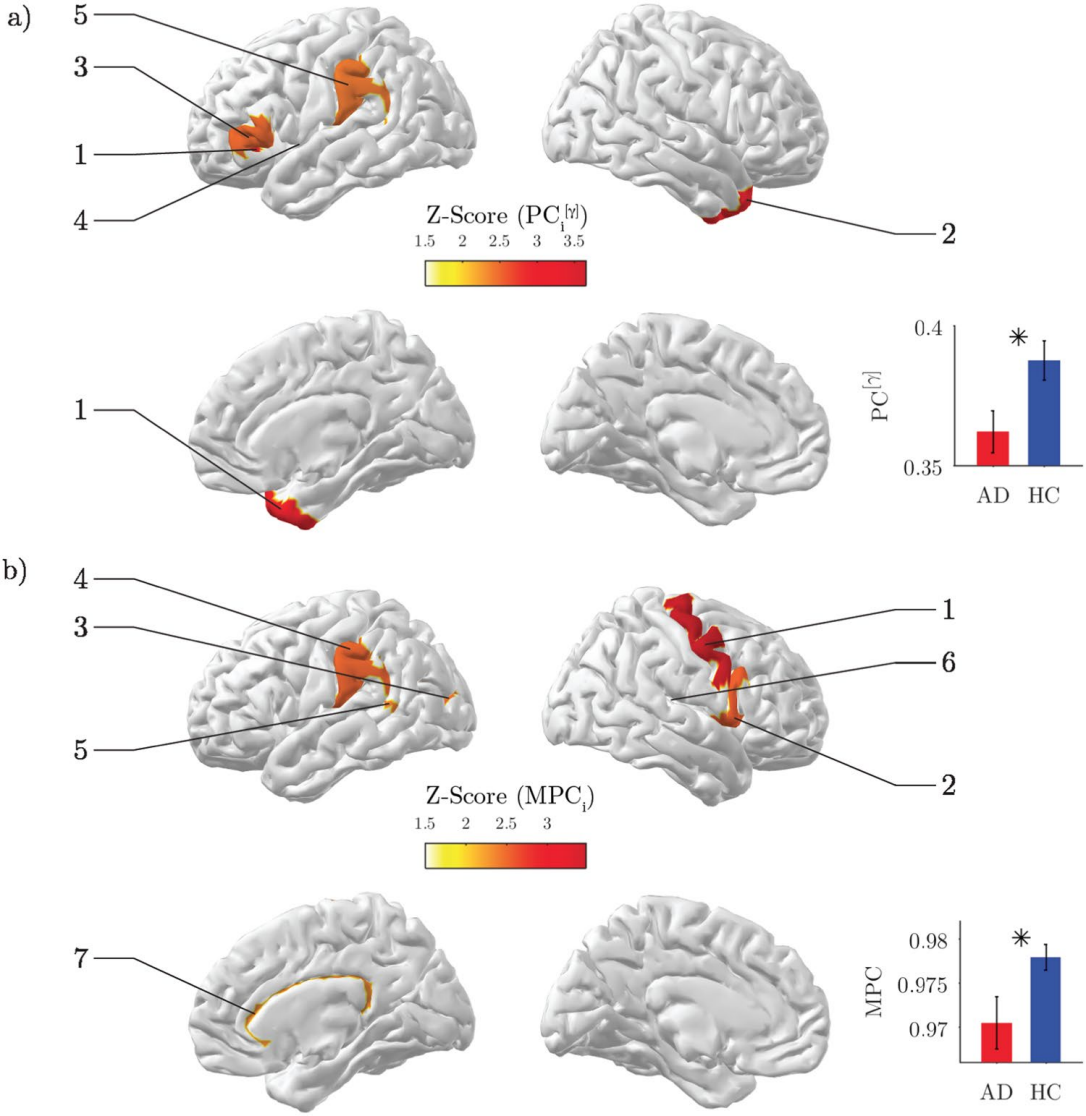
Network analysis of brain connectivity. (**a**) Inter-modular centrality. Statistical brain maps of group differences for local participation coefficients *PC*_*i*_ in the *gamma* band. Only significant differences are illustrated (*p* < 0.05, FDR corrected). The labels same ranks are used as labels. The inset shows the results for the global *PC*; vertical bars stand for group-averaged values while error bars denote standard error means. In both cases, Z-scores are computed using a non-parametric permutation t-test. b) Inter-frequency centrality. Statistical brain maps of group differences for local multi-participation coefficients *MPC*_*i*_. The inset shows the results for the global *MPC*; same conventions as in (**a**).

We first evaluated the results from the single-layer analysis. By inspecting the global participation coefficient *PC*, we reported in the *gamma* band a significant decrease of inter-modular connectivity in AD as compared to HC (*Z* = −2.50, *p* = 0.017; Fig. 3a inset). This behavior was locally identified in association ROIs including temporal and parietal areas (*p* < 0.05, FDR corrected; Fig. 3a; Table 2). No other significant differences were reported in other frequency bands or in flattened brain networks (Fig. S1).

**Table 2:** Statistical group differences for local brain network features. ROI labels, abbreviated according to the Destrieux atlas, are ranked according to the resulting *p*-values.

Feature	Rank	ROI label	Cortex	*Z* score	*p*-value
$P{C}_{i}^{[\gamma ]}$	1	Lat_Fis-ant-Horizont L	Frontal	−3.6507	0.0007
	2	Pole_temporal R	Temporal	−2.8642	0.0063
	3	G_front_inf-Triangul L	Frontal	−2.4562	0.0198
	4	**S_temporal_transverse L**	Temporal	−2.3887	0.0207
	5	**G_pariet_inf-Supramar L**	Parietal	−2.3820	0.0222
*MPC*_*i*_	1	G_precentral R	Motor	−3.4735	0.0006
	2	G_front_inf-Opercular R	Motor	−2.5239	0.0127
	3	S_oc_middle_and_Lunatus L	Occipital	−2.4582	0.0138
	4	**G_pariet_inf-Supramar L**	Parietal	−2.4860	0.0142
	5	**S_interm_prim-Jensen L**	Parietal	−2.3708	0.0147
	6	**S_temporal_transverse R**	Temporal	−2.3996	0.0191
	7	**S_pericallosal R**	Limbic	−2.3041	0.0203

The same ranks are used as labels in Fig. 3. ROIs highlighted in bold belong to the default mode network (DMN).

### Disrupted inter-frequency hub centrality

Then we assessed the results from the multi-layer analysis. Both AD and HC subjects exhibited high global multi-participation coefficients (*MPC* > 0.9), suggesting a general propensity of brain regions to promote interactions across frequency bands. However, such tendency was significantly lower in AD than HC subjects (*Z* = −2.24, *p* = 0.028; Fig. 3b inset). This loss of inter-frequency centrality was prevalent in association ROIs including temporal, parietal and cingulate areas, and with a minor extent in motor areas (*p* < 0.05, FDR corrected; Fig. 3b; Table 2).

**Figure 4:**
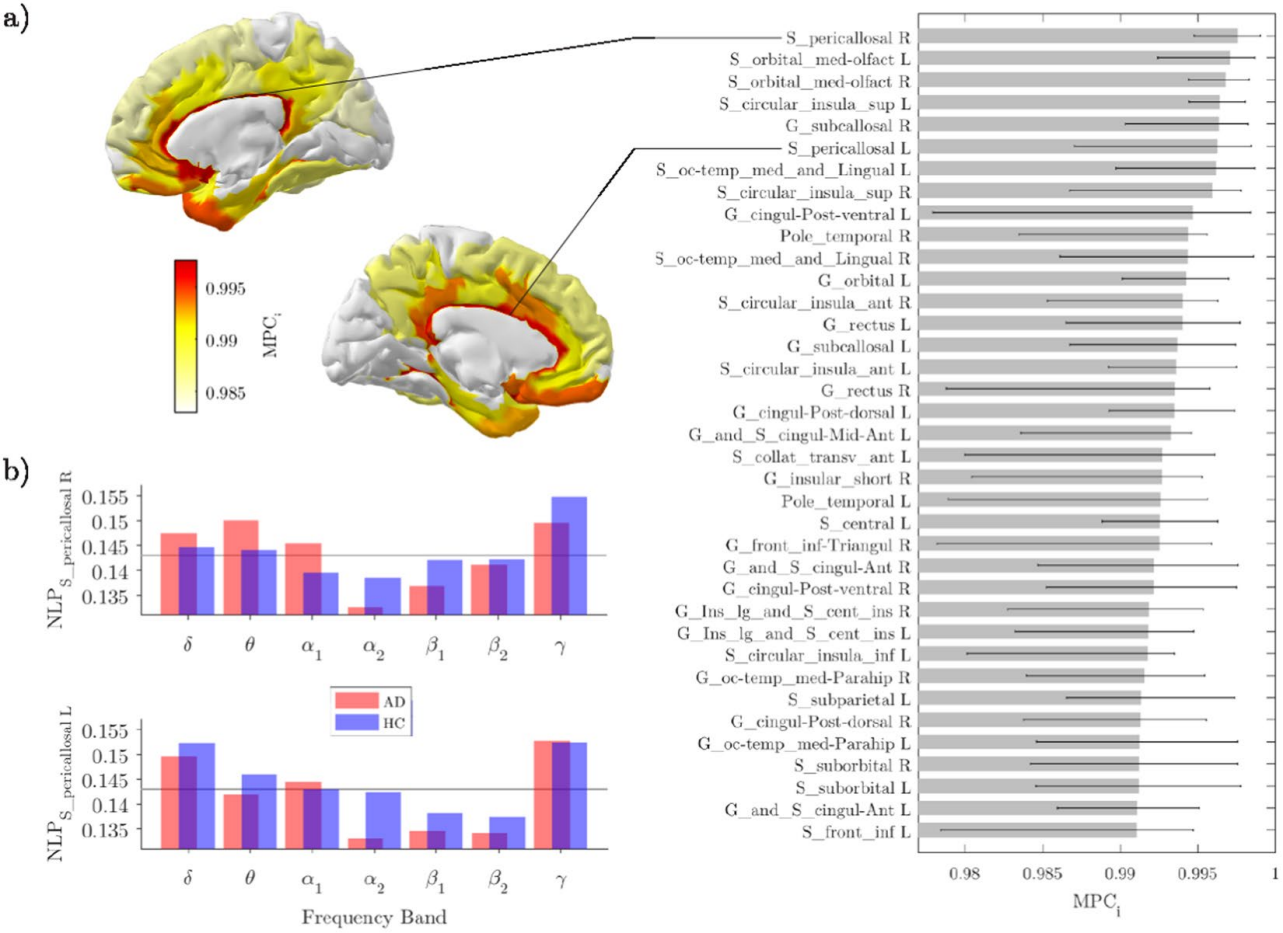
Inter-frequency hub centrality distribution. (**a**) The median values of local multi-participation coefficients (*MPC*_*i*_) are shown over the cortical surface for the healthy group. Only the top 25% is illustrated for the sake of visualization. The corresponding list of ROIs is illustrated in the horizontal bar plot. (**b**) Group-median values of the node-degree layer proportion (*NLP*_*i*_) for the right and left cingulate cortex. The grey line corresponds to the expected value if connectivity were equally distributed across frequency bands (*NLP*_*i*_ = 1/7).

Among those regions, the right cingulate cortex was classified as the main inter-frequency hub as revealed by the spatial distribution of the top 25% *MPC* values in the HC group (Fig. 4a). In HC subjects the connectivity of this region across bands, as measured by the node degree layer proportion *NLP*, was relatively stable (Kruskal-Wallis test, *χ* ^2^ = 10.79, *p* = 0.095), while it was significantly altered in AD subjects (Kruskall-Wallis test, *χ* ^2^ = 14.98, *p* = 0.020). In particular, the AD group exhibited a remarkably reduced *alpha*_2_ connectivity and increased *theta* connectivity (Fig. 4b). Similar results were also reported for the left cingulate cortex (AD: *χ* ^2^ = 11.89, *p* = 0.064; HC: *χ* ^2^ = 6.98, *p* = 0.323), although it was not significant in terms of *MPC* differences (Fig. 3b; Table 2).

### Diagnostic power of brain network features

**Figure 5:**
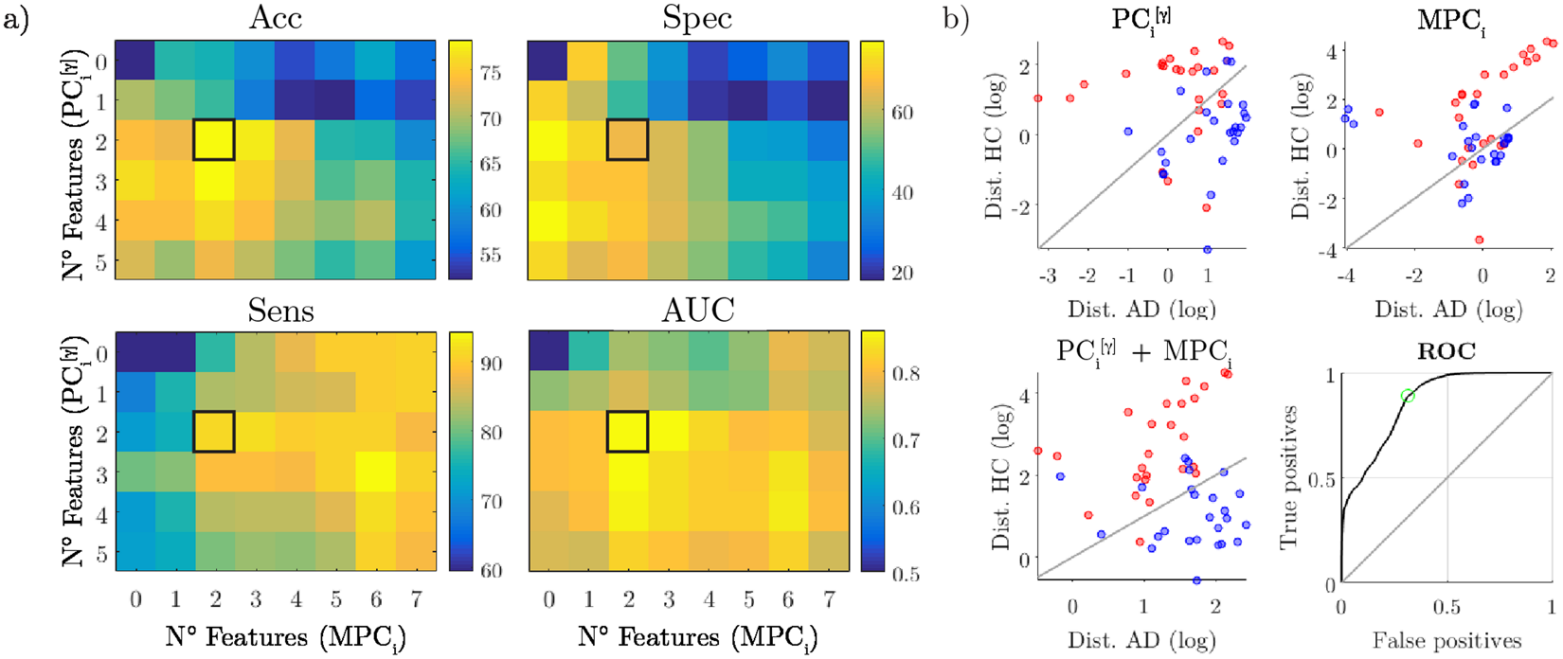
Classification performance of brain network features. (**a**) Matrices show the classification rates (accuracy = Acc, specificity = Spec, sensitivity = Sens, area under the curve = AUC) corresponding to the combination of the most significant $P{C}_{i}^{[\gamma ]}$ and *MPC*_*i*_ network features, respectively on the rows and columns of each matrix. Black squares highlight the highest accuracy rate and the corresponding specificity, sensitivity and AUC. (**b**) Scatter plots show the Mahalanobis distance of each subject from the *AD* and *HC* classes. Separation lines (*y* = *x*: equal distances) are drawn in grey. Red circles stand for Alzheimer’s disease (AD) subjects, blue ones for healthy controls (HC). The bottom right plot shows the ROC curve associated with the best network features configuration. The optimal point is marked by a green circle.

We adopted a classification approach to evaluate the power of the most significant local network properties in determining the state (i.e., healthy or diseased) of each individual subject. The best results were achieved neither when we considered single-layer features (i.e., $P{C}_{i}^{[\gamma ]}$) nor when we considered multi-layer features (*MPC*_*i*_) (respectively, first column and row of panels in Fig. 5a). Instead, a combination of the two most significant features gave the best classification in terms of accuracy (*Acc* = 78.39%) and area under the curve (*AUC* = 0.8625) (Fig. 5a,b). While the corresponding specificity was not particularly high (*Spec* = 65.68%), the sensitivity was remarkably elevated (*Sens* = 91.11%).

### Relationship with cognitive and memory impairment

**Figure 6:**
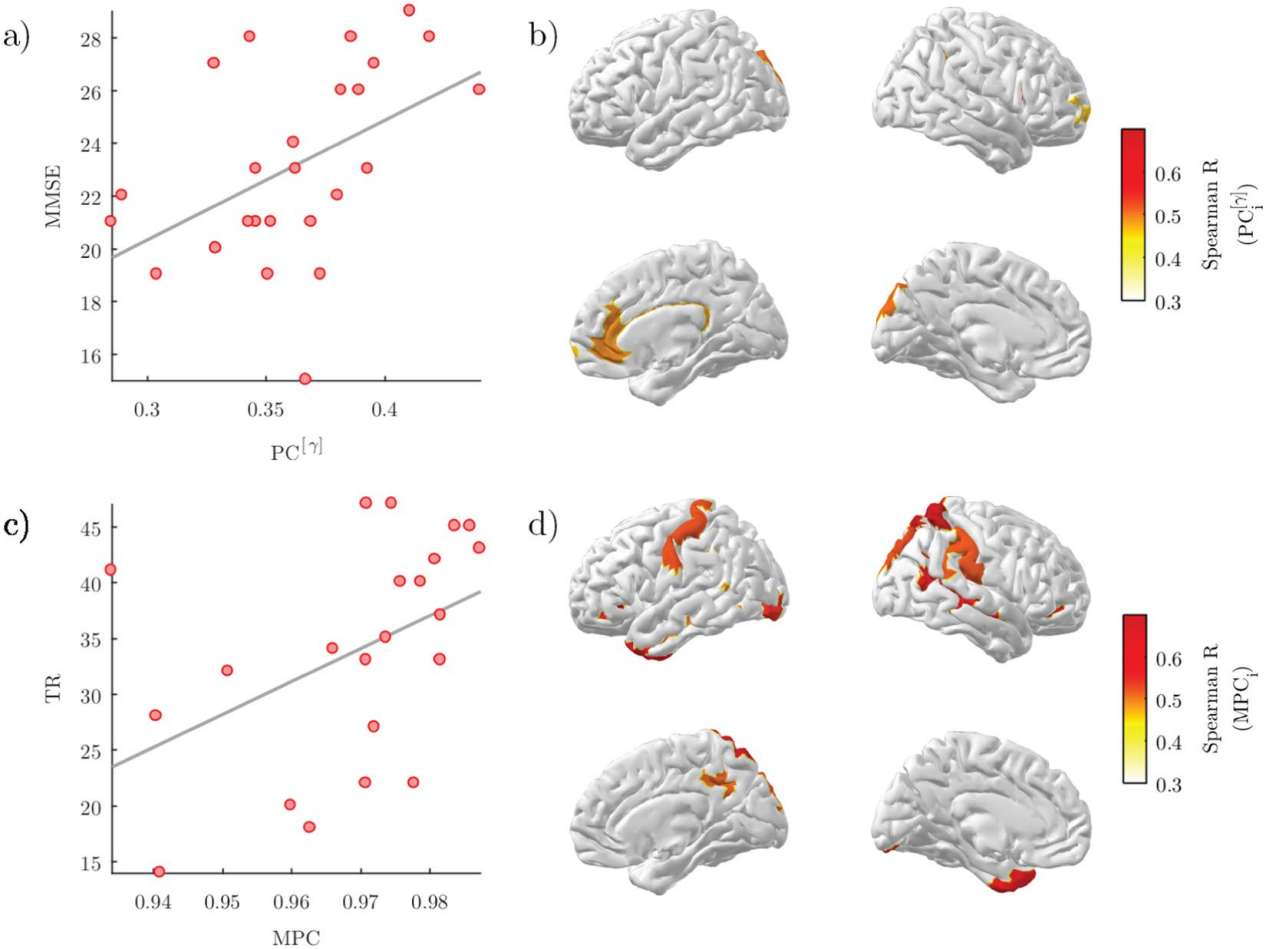
Correlation between brain network properties and cognitive/memory scores. (**a**) Scatter plot of the global participation coefficient in the *gamma* band (*PC* ^[*γ*]^) and the mini-mental state examination (MMSE) score of AD subjects (Spearman’s correlation *R* = 0.4909, *p* = 0.0127). (**b**) Correlation brain maps of the local participation coefficient in the *gamma* band ($P{C}_{i}^{[\gamma ]}$) and the mini-mental state examination (MMSE) score of AD subjects. Only significant *R* values are illustrated (*p* < 0.05, FDR corrected). (**c**) Scatter plot of the global multi-participation coefficient (*PC*) and the total recall (TR) score of AD subjects (Spearman’s correlation *R* = 0.5547, *p* = 0.0074). (**d**) Correlation brain maps of the local multi-participation coefficient (*MPC*_*i*_) and the total recall (TR) score of AD subjects. Only significant *R* values are illustrated (*p* < 0.05, FDR corrected).

We finally evaluated the ability of the significant brain network changes to predict the cognitive and memory performance of AD subjects. We first considered the results from single-layer analysis. We found a significant positive correlation between the global participation coefficient *PC* in the *gamma* band and the MMSE score (*R* = 0.4909, *p* = 0.0127; Fig. 6a). Then we considered the results from multi-layer analysis. We reported a higher significant positive correlation between the global multi-participation coefficient *MPC* and the TR score (*R* = 0.5547, *p* = 0.0074; Fig. 6c). These relationships were locally identified in specific ROIs including parietal, temporal and cingulate areas of the default mode network (DMN)^60^ (*p* < 0.05, FDR corrected; Fig. 6b,d; Table 3).

**Table 3:** Correlations of local brain network features and cognitive/memory scores.

Correlation	Rank	ROI label	Cortex	*R* coeff.	*p*-value
$P{C}_{i}^{[\gamma ]}$ - MMSE	1	Lat_Fis-ant-Vertical R	Frontal	0.5480	0.0046
	2	G_occipital_sup L	Occipital	0.5005	0.0108
	3	**S_interm_prim-Jensen R**	Parietal	0.4948	0.0119
	4	**G_and_S_cingul-Ant R**	Limbic	0.4864	0.0137
	5	**S_pericallosal R**	Limbic	0.4735	0.0168
	6	G_and_S_transv_frontopol R	Frontal	0.4585	0.0212
*MPC*_*i*_ - TR	1	Lat_Fis-ant-Horizont L	Frontal	0.6915	0.0004
	2	S_collat_transv_post L	Occipital	0.6706	0.0006
	3	S_circular_insula_ant L	Frontal	0.6214	0.0020
	4	**G_parietal_sup R**	Parietal	0.6061	0.0028
	5	S_orbital_lateral R	Frontal	0.5920	0.0037
	6	Pole_temporal L	Temporal	0.5739	0.0052
	7	S_orbital_lateral L	Frontal	0.5462	0.0085
	8	**S_temporal_sup R**	Temporal	0.5457	0.0086
	9	G_and_S_occipital_inf L	Occipital	0.5368	0.0100
	10	G_occipital_sup R	Occipital	0.5208	0.0130
	11	G_postcentral L	Sensory	0.5191	0.0133
	12	**G_pariet_inf-Supramar R**	Parietal	0.5151	0.0142
	13	**S_subparietal R**	Parietal	0.5066	0.0161
	14	**S_interm_prim-Jensen L**	Parietal	0.4915	0.0202
	15	**S_temporal_inf L**	Temporal	0.4869	0.0216

ROI labels, abbreviated according to the Destrieux atlas, are ranked according to the resulting *p*-values. ROIs written in bold belong to the default mode network (DMN).

## Discussion

Graph analysis of brain networks have been largely exploited in the study of AD with the aim to extract new predictive diagnostics of disease progression. Typical approaches in functional neuroimaging, characterized by oscillatory dynamics, analyze brain networks separately at different frequencies thus neglecting the available multivariate spectral information. Here, we adopted a method to formally take into account the topological information of multi-frequency connectomes obtained from source-reconstructed MEG signals in a group of AD and healthy subjects during EC resting states.

Main results showed that, while flattening networks of different frequency bands attenuates differences between AD and HC populations, keeping the multiplex nature of MEG connectomes allow to capture higher-order discriminant information. AD subjects exhibited an aberrant multiplex brain network structure that significantly reduced the global propensity to facilitate information propagation across frequency bands as compared to HC subjects (Fig. 3b, inset). This could be in part explained by the higher variability of the individual node degrees across bands (Fig. S2).

Such loss of inter-frequency centrality was mostly localized in association areas as well as in the cingulate cortex (Fig. 3b; Table 2), which resulted the most important hub promoting interaction across bands in the HC group (Fig. 4a). Because all these areas are typically affected by AD atrophy^4^ we hypothesize that the anatomical withering might have impacted the neural oscillatory mechanisms supporting large-scale brain functional integration. Notably, the significant alteration of the connectivity across bands observed in the cingulate cortex could be ascribed to typical M/EEG connectivity changes observed in AD, such as reduced *alpha* coherence^57–59, 61^ (Fig. 4b). We also found a significant decrease in the primary motor cortex (right precentral gyrus). While previous studies have identified this specific region as a connector hub in human brain networks^2^, its role in AD still needs to be clarified in terms of node centrality’s changes with respect to healthy conditions.

While flattening network layers represents in general an oversimplification, analyzing single layers can still be a valid approach that is worth of investigation. Because the *MPC* is a pure multiplex quantity, we considered the conceptually akin version for single-layer networks, the standard participation coefficient *PC*, which evaluates the tendency of nodes to integrate information from different modules, rather than from different layers^28, 47^. AD patients exhibited lower inter-modular connectivity in the *gamma* band with respect to HC subjects (Fig. 3a; Table 2) that was localized in association areas including frontal, temporal, and parietal cortices (Fig. 3a; Table 2). Damages to these regions can lead to deficits in attention, recognition and planning^62^. Our results support the hypothesis that AD could include a disconnection syndrome^63–65^. Furthermore, they are in line with previous findings showing *PC* decrements in AD, although those declines were more evident in lower frequency bands and therefore ascribed to possible long-range low-frequency connectivity alteration^2, 15^.

Put together, our findings indicated that AD alters the global brain network organization through connection disruption in several association regions (Figs 3a and 4a). In particular, we showed that the global loss of inter-modular interactions in the *gamma* band significantly affected the memory performance of AD patients as measured by the MMSE (Fig. 4a). These results suggest that the capacity of association areas to integrate information from other cortical regions through high-frequency channels, a crucial mechanism for sensory processing and memory retrieval^66–70^, becomes critically compromised in AD patients. Interestingly, such loss was paralleled by a diffused decrease of inter-frequency centrality. Future studies, involving recordings of limbic structures and/or stimulation-based techniques, should elucidate whether these two distinct reorganizational processes are truly independent or linked through possible cross-frequency mechanisms which are known to be essential for normal memory formation^71–73^.

As a confirmation of the complementary information carried out by the multi-layer approach, we reported an increased classification accuracy when combining the local *PC* and *MPC* features. The observed diagnostic power is in line with previous accuracy values obtained with standard graph theoretic approaches (around 80%) but exhibits slightly higher sensitivity (>90%), which is often desired to avoid false negatives^74–78^. Other approaches should determine if and to what extent the use of more sophisticated machine learning algorithms, or the inclusion of basic connectivity features^79–81^ and different imaging modalities^82^, can lead to higher classification performance and better diagnosis^2^.

Previous works have documented relationships between brain network properties and neuropsychological measurements in AD, suggesting a potential impact for monitoring disease progression and for the development of new therapies^7, 8, 10, 75, 83, 84^. This is especially true for the standard *PC* which has exhibited stronger correlations and larger between-group differences^2^. In line with this prediction, we also reported significant correlations between the MMSE cognitive scores and the *PC* values of the AD patients in the *gamma* band (Fig. 6a). An even stronger correlation was found, however, for the global *MPC* values and the TR scores (Fig. 6b, Table 3). Recent studies suggest that TR scores could be more specific for AD^85, 86^ as compared to MMSE scores which could be biased by differences in years of education, lack of sensitivity to progressive changes occurring with AD, as well as fail in detecting impairment caused by focal lesions^87^. Locally, the regions whose *MPC* correlated with TR were part of the default-mode network (DMN) (Table 3), which is heavily involved in memory formation and retrieval^60, 88^. According to recent hypothesis, these areas are directly affected by atrophy and metabolism disruption, as well as amyloid-*β* deposition^89, 90^.

Put together, our results suggest that AD symptoms related to episodic memory losses could be determined by the lower capacity of strategic DMN association areas to let information flow across different frequency channels. These results are in line with a recent study that adopted a similar multi-frequency network approach^91^, but that, however, *i)* did not perform a direct comparison with standard single-frequency network measurements and, more importantly, *ii)* did not provide a possible interpretation of the MPC in terms of its ability to favor communication across frequencies.

### Methodological considerations

As in many other biological systems, brain networks can be only inferred from experimentally obtained data^92, 93^. Hence, the resulting network only represents an estimate of the true underlying connectivity. In our study, MEG connectivity could be specifically influenced by linear mixing due to field spread effects (i.e., primary leakage) as well as by spurious interactions between areas spatially close to truly connected regions (i.e., secondary leakage)^91, 94^.

Here, we estimated brain networks by means of spectral coherence, a functional connectivity measure widely used in the electrophysiological literature because of its simplicity and relatively intuitive interpretation^95^. While this measure, as any other existing ones, cannot solve the problem of primary and secondary leakage effects, recent evidence showed that source reconstruction techniques, like the one we adopted here, can *i)* mitigate this bias^96, 97^, *ii)* generate connectivity patterns consistent within and between subjects^98^, and *iii)* help the interpretation of results in terms of cortical regions^97^.

To validate the obtained results we used, in a separate analysis, the imaginary coherence as a further approach to diminish field spread effects, at the cost, however, of removing possibly existing true interactions at zero-phase lag^94, 96, 99^. We demonstrated that while no significant between-group differences could be obtained in terms of *MPC* (data not shown here), the spatial distribution of the *MPC* values was very similar to that observed in brain networks obtained with the spectral coherence, especially for the internal regions along the longitudinal fissure (Fig. S3). Although, this is not a proof that we recovered true connectivity, it nevertheless validates the stability of our main results in terms of *MPC*.

Differently from other multiplex network quantities, such as those based on paths and walks^50^, the *MPC* has the advantage to not depend on the weights of the inter-layer links which, in general, are difficult to estimate or to assign from empirically obtained biological data. This is especially true in network neuroscience where, so far, the strength of the inter-layer connections is parametric and subject to arbitrariness^27^ or estimated through measures of cross-frequency coupling^21^ whose biological interpretation remains still to be completely elucidated^20^.

## Conclusions

We proposed a multi-layer network approach to characterize multi-frequency brain networks in Alzheimer’s disease. The obtained results gave new insights into the neural deterioration of Alzheimer’s disease by revealing an abnormal loss of inter-frequency centrality in memory-related association areas as well as in the cingulate cortex. Longitudinal studies, including prodromal mild cognitive impairment subjects, will need to assess the predictive value of this new information as a potential non-invasive biomarker for neurodegenerative diseases.

## Electronic supplementary material

Supplementary Material
