# Supplementary material for: Loss of brain inter-frequency hubs in Alzheimer's disease
Source: Sci Rep. 2017 Sep 7;7:10879. doi: 10.1038/s41598-017-07846-w (PMC5589939; doi:10.1038/s41598-017-07846-w)
Supplement: Supplementary file 1 — Supplementary Material [file 41598_2017_7846_MOESM1_ESM.pdf]

Loss of brain inter-frequency hubs in Alzheimer's disease

J. Guillon, Y. Attal, O. Colliot, V. La Corte, B. Dubois, D. Schwartz, M. Chavez, F. De Vico Fallani*

Supplementary Material

Supplementary Text

Coherency between signals

For a given frequency f, the coherency for the channels pair (i, j) can be computed as follow:

$$C_{ij}(f) = \frac{S_{ij}(f)}{\sqrt{S_{ii}(f)S_{jj}(f)}} \tag{S1}$$

where $S_{ij}(f)$ is the cross-spectrum of two time series $x_i(t)$ and $x_j(t)$ of ROI i and j respectively. Then the *spectral (or magnitude) coherence* is obtained by taking the absolute value of the coherency; and the *imaginary coherence* corresponds to the imaginary part of coherency. We used the same FFT parameters as for the PSD (section Methods).

Coefficient of variation

The global coefficient of variation is given by averaging CV_i values across all the nodes:

$$CV = \frac{1}{n}\sum_{i=1}^{N} CV_i = \frac{1}{n}\sum_{i=1}^{N} \frac{\sigma_{k_i}^{[\cdot]}}{k_i^{[\cdot]}} \tag{S2}$$

where $\sigma_{k_i}^{[\cdot]}$ is the standard deviation of the degree of node i across layers and $k_i^{[\cdot]}$ is the mean value.

Differently from MPC, CV tends to 0 when the links of the nodes tend to evenly distribute across layers, and give higher values when they rather tend to be concentrated in one layer or, more in general, differently distributed across layers.

*Corresponding author.
Email fabrizio.devicofallani@gmail.com

a)

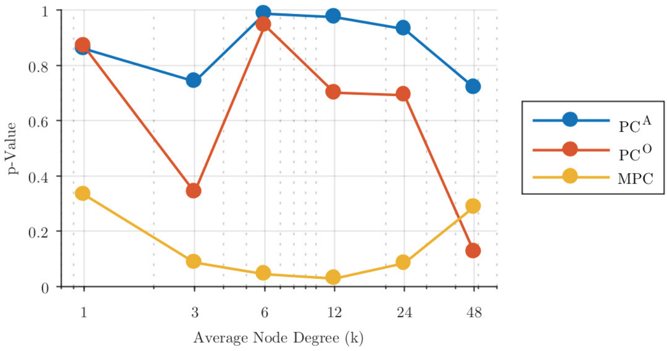

b)

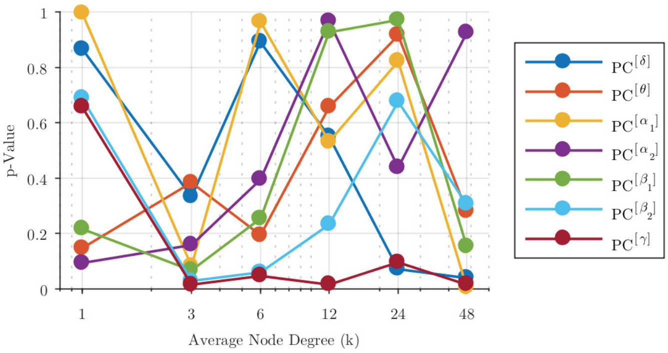

Figure S1: Statistical differences between global brain network properties of AD and HC subjects. These figures illustrate the p-values resulting from the permutation t-tests as a function of the average node degree k used to threshold the layers of the multi-frequency brain networks. In panel a), we show the p-values for multi-layer and flattened analysis whereas in panel b) the p-values resulting from single-layer analysis.

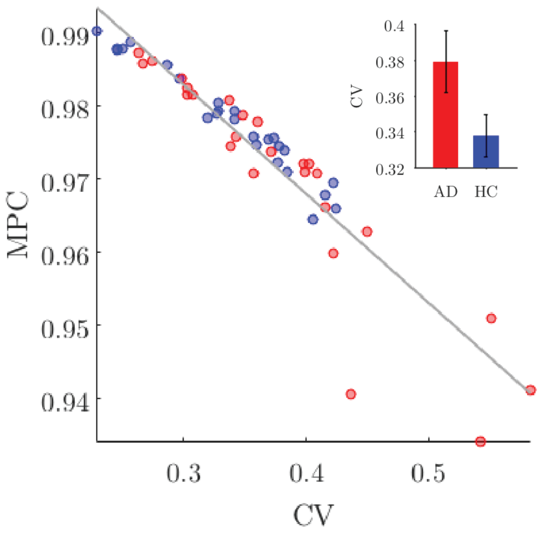

Figure S2: This figure shows the global coefficient of variation (CV): first the difference between the populations as an inset plot ($p = 0.0521$) and the correlation with the global multi-participation coefficient (MPC) as a main plot ($p < 10^{-15}$, $R = -0.9742$).

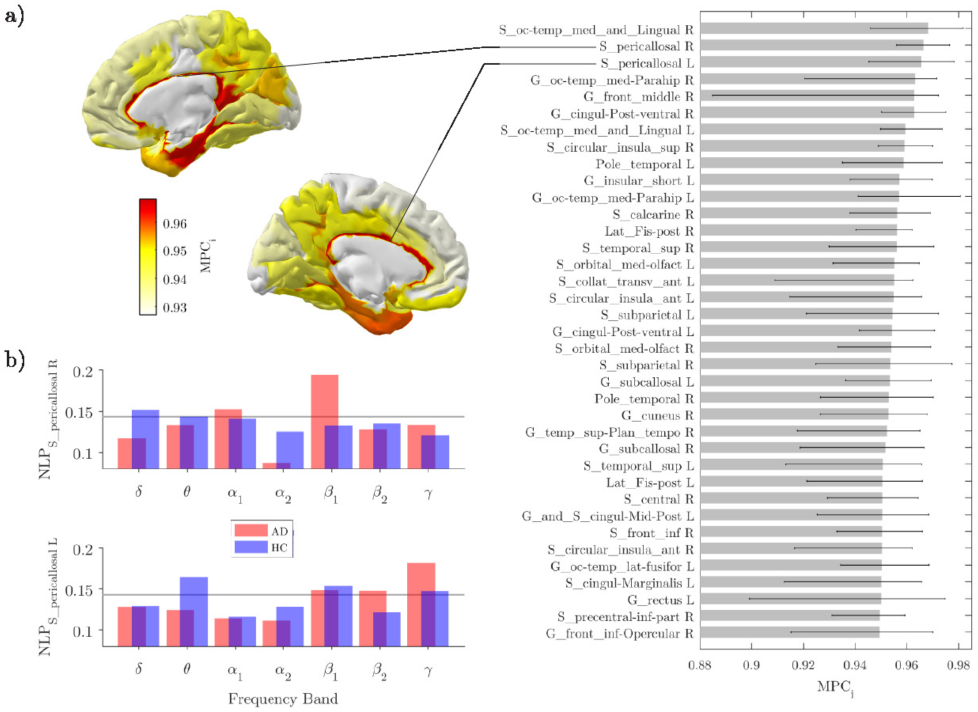

Figure S3: Inter-frequency hub centrality distribution for brain networks obtained with imaginary coherence. a) The median values of local multi-participation coefficients (MPC_i) are shown over the cortical surface for the healthy group. Only the top 25% is illustrated for the sake of visualization. The corresponding list of ROIs is illustrated in the horizontal bar plot. b) Group-median values of the node-degree layer proportion (NLP_i) for the right and left cingulate cortex. The grey line corresponds to the expected value if connectivity were equally distributed across frequency bands ($NLP = 1/7$).
